# Combined Distributed Shared-Buffered and Diagonally-Linked Mesh Topology for High-Performance Interconnect

**DOI:** 10.3390/mi13122246

**Published:** 2022-12-17

**Authors:** Charles Effiong, Gilles Sassatelli, Abdoulaye Gamatié

**Affiliations:** LIRMM, University of Montpellier, CNRS, 34000 Montpellier, France

**Keywords:** network-on-chip, buffers, resource sharing, energy efficiency, adaptive control

## Abstract

Networks-on-Chip (NoCs) have become the *de-facto* on-chip interconnect for multi/manycore systems. A typical NoC router is made up of buffers used to store packets that are unable to advance to their desired destination. However, buffers consume significant power/area and are often underutilized, especially in cases of applications with non-uniform traffic patterns thus leading to performance degradation for such applications. To improve network performance, the Roundabout NoC (*R-NoC*) concept is considered. *R-NoC* is inspired by real-life multi-lane traffic roundabouts and consists of lanes that are shared by multiple input/output ports to maximize buffering resource utilization. *R-NoC* relies on router-internal adaptive routing that decides the lane path based on back pressure. Back pressure makes it possible to assess lane utilization and route packets accordingly. This is made possible thanks to the use of elastic buffers for control flow, a clever type of handshaking in a way similar to asynchronous circuits. Another prominent feature of R-NoC is that internal routing and arbitration are completely distributed which allows for significant freedom in deciding internal router topology and parameters. This work leverages this property and proposes novel yet unexplored configurations for which an in-depth evaluation of corresponding implementations on 45 nm CMOS technology is given. Each configuration is evaluated performance and power-wise on both synthetic and real application traffic. Several *R-NoC* configurations are identified and demonstrated to provide very significant performance improvements over standard mesh configurations and a typical input-buffered router, without compromising area and power consumption. Exploiting the distributed nature of *R-NoC* routers, a diagonally-linked configuration is then proposed which incurs moderate area overhead and features yet better performance and energy efficiency.

## 1. Introduction

As the number of IP cores grows in a System-on-Chip, traditional on-chip interconnects, such as the bus and crossbar cannot meet the performance, energy efficiency, and scalability demands of many core systems [1]. Networks-on-Chip (NoCs) is a viable substitute interconnect template providing enhanced scalability and performance thanks to path diversity [1]. NoCs are composed of routers interconnected by point-to-point data links. Routers play the crucial role of routing packets from one node to the other in the network. A typical NoC router is composed of input and/or output buffers. These buffers help mitigate contentions by means of temporarily storing unit data chunks (packets or flits) that cannot advance to their destination, thereby limiting the spillover effect on the routing links that remain available for conveying data. Buffers play an important role in high-performance systems in which a number of processing engines go by the thousand in most advanced designs [1].

A router buffer is shown to be the most expensive resource, consuming significant area and power in the network [2]. It however appears that they are often underutilized, especially in the case of non-uniform traffic patterns [2]. Indeed, usual NoC routers have static port-allocated buffers (port-pinning), which implies that any thru-traffic can use only a fraction of the router buffer resources.

The buffer under-utilization issue has been quantified in [3], where the authors described a scenario based on a (8×8)-mesh network composed of input-buffered routers. The network has been simulated for 30,000 clock cycles while considering both uniform and non-uniform (i.e., transpose and bit-complement) traffic patterns. It was observed that only about 10% of the buffering resources were unutilized on average i.e., empty in the case of uniform traffic. Non-uniform traffic patterns experiments however showed that 45% to 47.5% of the total NoC buffers remained idle, revealing a massive under-utilization. This suggests that the NoC is not only under-performing w.r.t. the buffering resources it actually possesses, but this also worsens power efficiency as pointed out in aforementioned studies [2,3].

The *Roundabout-NoC (R-NoC)* concept, dedicated to mesh network topologies [4,5] was proposed with high buffering resources utilization as the primary design objective. *R-NoC* is inspired by real-life traffic roundabouts and consists of lanes, across which buffers are distributed, and shared by multiple input/output ports for efficient traffic management. Owing to its inspiration from real-life traffic roundabouts, internal routing and arbitration are distributed and adaptive. It shares similar *ring-like/traffic-roundabout* features to the popular Rotary router [6]. However, as it is extremely challenging to use wormhole flow-control in ring-like router architectures (or folded network topologies like Torus), the Rotary router [6] uses combined *virtual-cut-through (VCT) and bubble flow-controls*, known as *local bubble scheme*, to circumvent deadlocks occurrence. Unfortunately, VCT requires large buffers, which introduce overheads in terms of area and power and therefore impede applicability for on-chip communication networks.

In this paper, we show that the initial *R-NoC* template can easily be extended to non-mesh topologies by means of devising a diagonal-mesh topology. Compared to the basic mesh network topology, the *R-NoC-D* based diagonally-linked mesh topology provides shorter network paths and reduces network congestion [7]. In addition, as it is shown in our experiments, faster data transfers contribute to reducing the dynamic power consumption due to reduced switching activities in the network.

The main contributions of this paper can be summarized as follows:

- We elaborate on the initial concept, in particular focusing on the freedom offered by *R-NoC* in terms of internal topology, the opportunity for concentrated networks, and the number of ports. We propose a construction algorithm that generates deadlock-free router topologies taking design parameters as inputs.
- We present in detail the synchronous-elastic implementation of *R-NoC* and thoroughly evaluate the corresponding power consumption at the network level while considering different traffic patterns. We show that *R-NoC* consumes less power compared to typical input-buffered routers. This confirms our claim that *R-NoC* performance improvement is not at the expense of power.
- We investigate how the network performance scales with an increasing number of cores, from 4 to 64 cores, and for various traffic patterns. The obtained experimental results show that *R-NoC* provides better scalability compared to typical input-buffered routers.
- We propose a low area-overhead router, named *R-NoC-D*, dedicated to DMesh networks. We show that the new router provides up to 24% and 59% performance improvement for uniform and non-uniform traffic patterns respectively compared to its mesh counterparts. We assess the implementation of *R-NoC-D* and observe that the performance improvements it brings come at an area overhead that is 13.7% less compared to a DMesh implementation of a typical router with input buffers.

The remainder of this paper is organized as follows. Section 2 describes related works. *R-NoC* alongside the construction algorithm is introduced in Section 3, while its implementation is described in Section 4. Section 5 presents *R-NoC* evaluation, followed in Section 6 by the assessment of the proposed *R-NoC-D* configuration. Finally, Section 7 gives concluding remarks and perspectives.

## 2. Related Works

NoC literature is rather vast and in this section, we purposely do not present NoCs at large but rather quickly introduce the specifics of conventional packet-switching/wormhole-based NoC and focus the discussion on the distinctive features of *R-NoC* and the relevant proposals found in the literature for comparative purposes.

### 2.1. Conventional Packet-Switching Routers

While a vast number of improvements have been proposed since NoC introduction 2 decades ago [8,9], the conventional router microarchitecture is often pipelined, comprises a crossbar, and uses input or output ports. Allocating buffers to input ports is the most popular design style found in the NoC arena as it often performs better compared to output buffering, due to the opportunity to buffer packets regardless of their router destination port. An example of a well-known typical input-buffered router is the Hermes router [10] which, despite not possessing a pipelined microarchitecture, represents otherwise fairly well-conventional NoC routers. The Hermes router is composed of input FIFO queues. A blocked packet (i.e., flits of a packet that cannot advance to the desired output ports) waits in queues until the output port is available [10]. A major drawback of that design decision is that the FIFOs are dedicated to the input ports and can only be exploited by data flows using the corresponding input ports. Conversely, *R-NoC* consists of lanes shared by multiple input/output ports. Hence, buffers are more effectively exploited for performance/energy gains regardless of application traffic patterns. The actual implementation of *R-NoC* however raises additional challenges among which the necessity of fine-grain backpressure from the head-of-line (HOL): whenever the first flit of a packet hits a barrier (forward resource busy), the entire pipeline must be stalled. This problem (even though heavily mitigated by the internal deflections *R-NoC* routers perform) happens to be adequately solved by means of using asynchronous or synchronous-elastic design, as discussed in Section 4.

### 2.2. Router-Less, Buffer-Less and Shared-Buffer Routers

In an attempt to mitigate area and power overheads associated with conventional NoCs, several recent works focused on drastically simplifying or conversely better using NoC resources. Router-less NoCs [11,12,13] have recently been proposed, based on eliminating routers (i.e., the routing function) and relying on design-time decided physical routes serving all IP the system is made of. This can further be combined with rather dense physical interconnects [14] that exploit advanced wiring capabilities of contemporary processes. Buffer-less [15] routers completely remove buffers inside routers. This reduces on-chip area overhead and saves a considerable amount of the total router power dissipation. In buffer-less routing, packets that are unable to progress to their desired destination ports are deflected or misrouted since there are no buffers in the router to store packets. Packets are continuously deflected from hop to hop until they finally reach their desired destination nodes. Intuitively, only a few packets are deflected at light traffic injection rates due to low network contentions. However, it has been observed that buffer-less routers consume significantly more dynamic power and have higher packet latency compared to buffered routers [2,16]. This is because more and more packets get deflected further away from their destination nodes at high traffic injection rates and the deflected packets unnecessarily consume the router link bandwidth. Buffer-less design style may therefore be a good fit for moderate-bandwidth (and low-latency) application domains with tight power constraints, but do not provide a satisfactory solution for traffic-intensive use cases.

On the other hand, shared-buffer routers are found in router architectures allowing the buffering resources to be shared by multiple input ports for performance benefits instead of dedicating a set of buffers to each input port. Such an architecture with distributed shared buffer was proposed in [17]. This router emulates output buffers and provides a higher throughput compared to typical input-buffered routers. However, performance improvement is at the expense of area/power overheads caused by an additional crossbar and arbitration strategy employed in the router. Conversely, *R-NoC* uses distributed buffering and improves performance without sacrificing area/power.

The RoShaQ [2] consists of buffering queues known as shared queues. Packets are allowed to use the queues when they are empty or if they have similar output destinations to avoid deadlocks. RoShaQ employs a bypass technique for the shared queues. This allows packets to travel from input to output ports without using the shared queue, thus reducing the no-load latency of the network. A drawback of the RoShaQ approach is that it requires an additional crossbar for allocating the shared queues. This introduces the additional area and power overheads in the router. *R-NoC* does not use explicit crossbar and input buffers. Hence, *R-NoC* avoids the additional area and power dissipation associated with crossbars and input buffers.

ViChaR [18] is a unified buffer structure in which buffer allocation is performed based on dynamically-allocated virtual channels (VCs). This is particularly efficient as VCs are allocated on-demand and buffering resources are shared among the same. Authors report 25% performance gains against an NoC having the same buffering resources, or conversely 50% buffer reduction with no impact on performance. *R-NoC* performs buffer allocation on the router-internal path level and requires no VC support. This process is finer grain and results from the internal deflections occurring within the router, within a few clock cycles.

**Figure 1:**
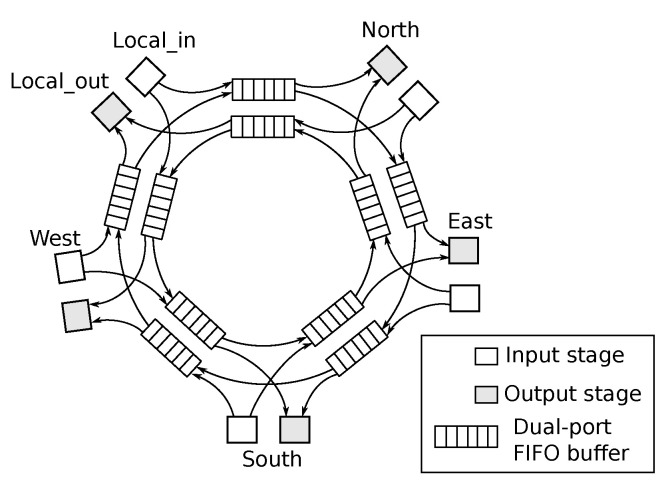
The Rotary router architecture.

**The Rotary router.** The Rotary [6] router concept is similar to R-NoC in terms of its internal traffic roundabout/ring-like architecture. A rotary router is made up of two independent rings constructed using Dual-port FIFO buffers (DBF) illustrated in Figure 1, which is inspired by the Rotary architecture [6]. In Rotary, the *input stage* logic decides which of the rings to route incoming packets to. At low load, incoming packets are forwarded depending on their nearness to a suited output port, while the ring occupancy is considered at medium to high loads. An incoming packet travels along the ring to its destined output port and exits the router if the output is available. Conversely, the packets continue along the ring until an alternative suitable port is determined.

A packet in Rotary [6] adaptively uses the first available output port after making a specified number of turns in the router. This strategy is intended to avoid head-of-line blocking since packets can proceed to the next DBF, thereby allowing packets that are behind to advance. The Rotary router [6] is topology agnostic and uses a low complexity adaptive routing. The Rotary router [6] relies on combined virtual-cut-through (VCT) and bubble flow controls to avoid deadlocks in the rings. VCT allocates buffers to an entire packet, thereby requiring large buffers in the router. Bubble flow control is used to control packet injection ensuring that packets can only be injected into the ring if there will exist two bubbles in the ring after injection.

Similarly, the *R-NoC* router uses distributed control and arbitration. *R-NoC* however uses wormhole flow control which places no constraint on the minimum buffer size (which must not accommodate an entire packer) and therefore reduces the router area and power overheads associated with large buffers while enhancing buffer utilization and reducing message latency. *R-NoC*, unlike the *Rotary*, uses a simpler livelock-free routing algorithm which is easy to implement in hardware, yet has the capability to adaptively deflect packets upon contention (lane switching). Another opportunity brought by the distributed nature of control and arbitration at the router level is the design of routers with an arbitrary number of ports. This is illustrated in this paper with the evaluation of diagonally meshed topologies having 9 ports, but can be extended to heterogeneous topologies which are considered in several recent works [14,19].

## 3. *R-NoC* Router for Resource Sharing

This section elaborates on the *R-NoC* concept. We describe the deadlock problem and present an algorithm for generating deadlock-free *R-NoC* router topologies. We illustrate the capabilities with discussions around several generated *R-NoC* router topologies. Here, we use *configuration* and *topology* interchangeably to refer to the manner in which the lanes are arranged in the router.

### 3.1. Intuition on the Data-Flow Principle in R-NoC Router

**Figure 2:**
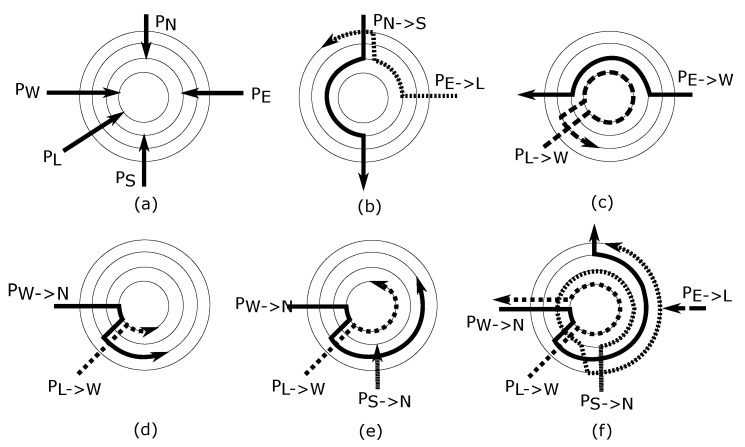
Packet flow scenarios in deadlock-free *R-NoC*: (**a**) Packet entry (**b**) Lane switching caused by blocked path (**c**) Lane switching when packet output port is busy. $P_{X\to Y}$: a packet from *X* input port and destined for *Y* output port. (**d**) Low traffic: a packet from the west switches lane and uses *secondary* lane resources. (**e**,**f**) More lane resources are being used.

The *R-NoC* router is inspired by real-life multi-lane traffic roundabouts where cars go on a lane and switch to high-priority lanes should they miss their exit (adaptive deflection). In doing so, deflected packets travel across lanes that possess buffers and are therefore buffered in a distributed manner. A direct implication of that principle is that packets tend to take a shorter route at low traffic (i.e., lower latency) and exploit additional lanes as traffic grows. Different packet flows are illustrated in Figure 2 for a deadlock-free *R-NoC* router. In the shown multi-lane router topologies, *primary* and *secondary* lanes are considered. *Primary* lanes can be attached to both input and output ports of the router, whereas *secondary* lanes can only be attached to output ports.

This is illustrated in Figure 2a where input ports are distributed only to the *primary* lanes as shown whereas *secondary* lanes are exploited only whenever congestion occurs. When a packet flowing on a *primary* lane is blocked or not granted access to the output port, it switches to a *secondary* lane via a *switch link*. A scenario, where a packet switches to a *secondary* lane because of a blocked path, is shown in Figure 2b. In Figure 2b, a packet from the east input port destined for the local output port switches to a *secondary* lane because the path is being used by another packet. This design choice avoids queuing packets on the lane (if possible) whenever multiple flows compete for shortest path resources, which increases router resource utilization and packet throughput. A side-benefit of this strategy lies in the variable router traversal latency which is minimal under light traffic and grows are *secondary* lanes participate to packet buffering. Figure 2c shows a scenario where a packet from the local input port and destined for the west output port switches to a *secondary* lane because the west output port is occupied by a packet flowing from the east input port to west output port. Packets can only make forward progress towards a *secondary* lane if the desired lane resource is available. Otherwise, they are queued on the lane. The output port is granted in a *round-robin* manner if two simultaneous requests for a given output port originate from either two *primary* or two *secondary* lanes. Note that all of these arbitration decisions are made in a distributed and lane-local fashion, as described in Section 4.

More generally, the main properties of *R-NoC* can be summarized as follows:

1. the router can have *N* lanes, where N≥2. The lanes are partitioned into *primary* and *secondary* lanes with an arbitrary lane count in each;
2. the output ports are connected to both *primary* and *secondary* lanes, while the input ports are only connected to the primary lanes;
3. packets can switch from *primary* to *secondary* lanes when either their path is blocked or their output is unavailable.

An incoming packet travels along the ring to its destined output port and exits the router if the output is available. Conversely, the packets continue along the ring until an alternative suitable port is determined, as shown through Figure 2d–f.

### 3.2. The R-NoC Router Concept

**Figure 3:**
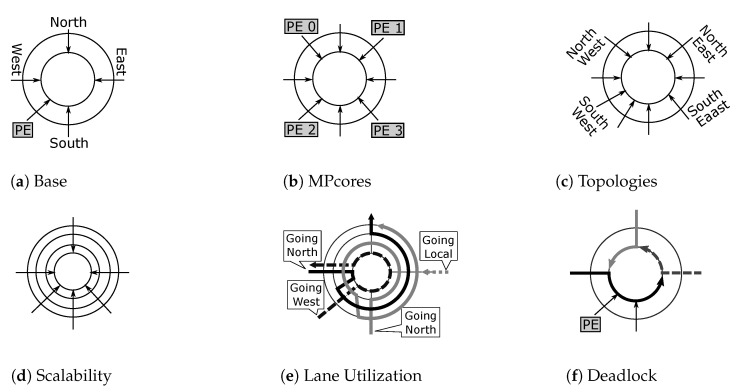
Initial deadlock-prone *R-NoC* topology and data-flows scenarios (**a**–**f**).

*R-NoC* provides a highly-adaptable architecture, which allows the router to be configured to meet numerous network topologies and applications demands. Figure 3a shows the router concept, where the available resources are inherently shared by multiple ports. All of the control, handshaking, and arbitration are performed in a purely distributed manner at the level of *port controllers* and *lane controllers* which are extensively described in Section 4. Thanks to the distributed nature of the control in *R-NoC*, varying the number of lanes, input ports, and output ports require very few modifications, granted certain assembling properties are met. The architecture can indeed be readily adapted for devising *concentrated networks*, in which more than one core is attached to a router. Figure 3b conceptually illustrates this property in which 4 cores are attached to a single lane that acts as a local interconnect. An example is the “X-Network” [20] that connects each router to four neighboring cores which have the benefit of area reduction and performance improvements. Similarly, an arbitrary number of input/output ports can be attached to the router (to the same or different lanes), which makes for a denser network topology as illustrated in Figure 3c).

*R-NoC* is further highly scalable in terms of the number of lanes, the number of buffers (that can be arbitrarily distributed), and configurable through deciding its internal topology that can be tuned to favor certain connections/routes if needed as illustrated in Figure 3d. Most of the router buffers become utilized when the network traffic is high (see Figure 3e whereas under light load only the innermost lanes get used as packets take shorter routes to their desired output ports. This proportional buffer utilization favors improved network performance and provides an opportunity for smart power management, where some outer lanes can be turned on only when needed (i.e., under high communication load), otherwise helping to reduce power consumption.

Sharing all of the lanes between each and every input port allows the router buffering resources to be adequately utilized, which significantly improves network throughput. Nevertheless, allowing packets from all input ports to use all of the lane resources can lead to deadlocks on the lanes. This is due to cyclic dependencies that arise when packets are simultaneously occupying some lane resources and requesting lane resources occupied by other packets [16]. An example of such a situation is depicted in Figure 3f.

### 3.3. Generating Deadlock-Free R-NoC Topologies

The flexibility granted by *R-NoC* in terms of routing comes at the expense of deadlock-proneness: intuitively a packet may be utilizing routing resources required by another packet for advancing to its destination. That other packet will be stalled and will not free up resources required by the first packet: this forms an interlock that cannot be resolved unless a packet is dropped.

The *R-NoC* topology shown in Figure 3a is deadlock-prone as cyclic dependencies can occur on the lanes. To avoid such issues, we previously sketched some ways to obtain deadlock-free *R-NoC* router configurations [5]. Here, we build an algorithm, which aims to formalize the systematic generation of deadlock-free *R-NoC* router topologies.

We first observe that achieving the expected deadlock-free routers requires a combination of input ports sharing a set of lane resources, without introducing cyclic dependencies in the router and the corresponding network. The input ports must also be distributed to the lane such that the router is not disconnected, i.e., valid paths must exist from input ports to output ports in the router. Algorithm 1 defines deadlock-free *R-NoC* topology generation through a number of steps as explained in the sequel. It offers a systematic process for the construction of deadlock-free *R-NoC* routers topologies. The generated topologies then require to be manually implemented using an arbitrary Hardware Description Language (HDL) as the tool does not generate synthesis-ready HDL. The algorithm operates as follows:

In Step 1, input data are declared. This algorithm takes a set of lanes (*L*), a set of input ports (*I*), and a set of output ports for a given input port ($O_{i}$) as inputs.

In Step 2, the sets required to store information about lanes and their shareable input ports are created.
**Algorithm 1:***R-NoC* topology generation algorithm
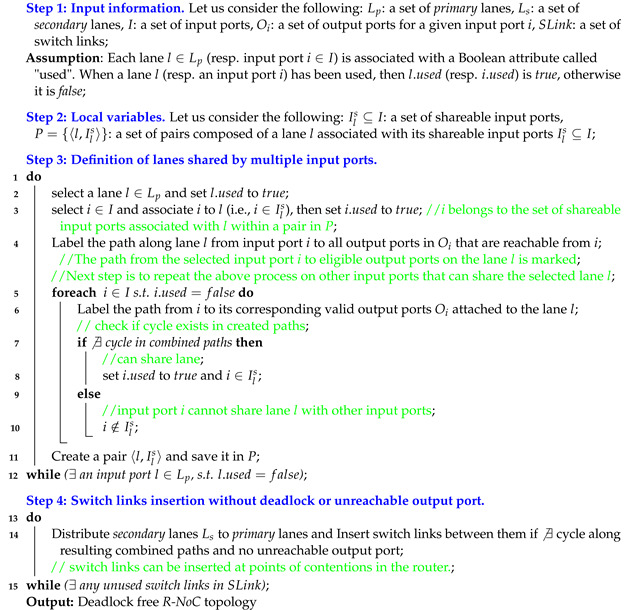


Step 3 represents the heart of the algorithm. A given lane *l* is selected from the set of lanes in *line 2*. *Line 3* selects an input port from the set of input ports and associates it with the selected lane. In *line 4*, the path from the selected input port to all output ports is marked. At this stage, no cycle exists in the lane since only one input port is associated with the selected lane. The next phase is to find other input ports that can share the selected lane *l* with the currently associated input port *i*. For this purpose, the algorithm loops through all the set of input ports in *line 5 to line 12*. In each iteration, an input port is selected and associated with the lane. The path from the selected input port (*i*) to all valid output ports ($O_{i}$) is marked in *line 6*. Then, the algorithm checks if a cycle exists in the lane *L*. If a cycle exists then the currently selected input port (*i*) cannot share the selected lane *l* resources with the other input port(s). On the other hand, if no cycle exists, then the currently selected input port can be shared with the other input port(s). The iteration is repeated (for all unused input ports) until all the input ports have been successfully attached to a lane.

Having identified input ports that can share the *primary* lanes, ***Step 4*** connects different lanes by inserting switch links across them: on the one hand, between *primary* and *secondary* lanes, and on the other hand, between *secondary* lanes depending on their number. The insertion of the switch links must be done carefully so that the links do not introduce cycles or unreachable output ports. For this reason, a switch link can be added if and only if its introduction will not lead to a deadlock or unreachable output port in the router. In order to formally assess the deadlock-freeness of the generated lanes and router, we use the well-known theoretical model for deadlock avoidance proposed by Duato [21] and Dally [16]. This model relies on building a *channel dependency graph* (CDG) of the shared network resources and identifying cyclic dependencies. Deadlock can potentially occur if cyclic dependencies exist between shared resources. Algorithm 1 assumes a dimension-order routing (DOR) or a quasi-minimal routing algorithm [7]. Also, generated *R-NoC* router configurations can only be plugged into deadlock-free NoCs, typically using deterministic or minimal-adaptive routing algorithms. This guarantees deadlock-freeness at the network level. The current version of *R-NoC* does not support fully adaptive routing as they are deadlock-prone.

### 3.4. Example of R-NoC Router Topology Generation

We show the application of Algorithm 1 on a concrete example. For this purpose, we assume a *R-NoC* router for a mesh network topology and *XY-routing* function. Table 1 gives the list of input ports and their valid output ports for *XY routing algorithm*. Thus, in ***step 1*** of Algorithm 1, the input information is a set of four lanes, a set of input ports, and a set of output ports for each input port according to Table 1. Next, ***step 3*** can be applied to devise the list of input ports that can share the lanes such that cyclic dependencies are avoided on the shared lanes.

**Figure 4:**
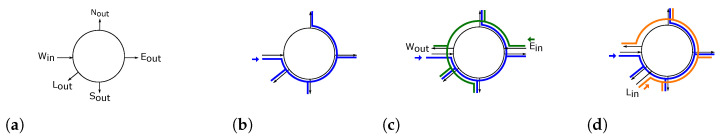
*R-NoC* router generation for mesh network. (**a**) West in. and possible out. ports. (**b**) Out. ports for west in. port. (**c**) Out. ports for west and east in. ports. (**d**) Out. ports for west and local in.

Assuming that the *west input port* is selected from the set of inputs port in *line 2* of Algorithm 1 and associated to a selected lane *l* in *line 3*, then the resulting configuration is shown in Figure 4a, where the west input port and its valid output ports are connected to the lane. Upon executing *line 4* of the algorithm, the resulting configuration is shown in Figure 4b with “blue label” indicating valid output ports for a packet flowing from the west input port as displayed in Table 1. No cyclic dependency exists in Figure 4b as the path between *north output port* to *west input port* is not labelled.

The next step is to determine the other input ports that can share the selected lane with the *west input port*. For this reason, assuming that *line 5* (first iteration) selects the *east input port*, executing *line 6* gives the configuration shown in Figure 4c. In Figure 4c, the lane has both blue and green labels. The green label represents valid output ports for packets flowing from the east input port. The next step is to check if a cycle exists in the lane. It is visible from the figure that both labels form a cycle on the lane (i.e., all the paths on the lanes have labels). Therefore, the *west input port* cannot share the selected lane resources with the *east input port*.

**Figure 5:**
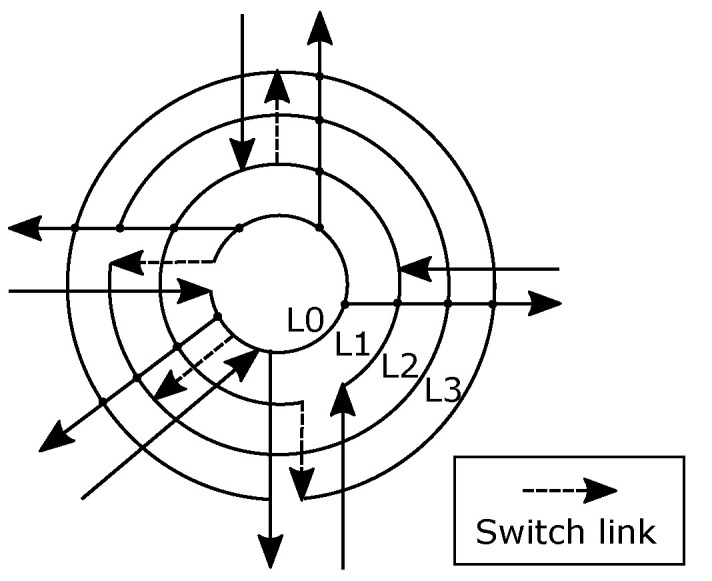
Generated *R-NoC* topology. L${}_{n}$: Lane 0–3.

The control flow of Algorithm 1 goes back to *line 5* to find possible input ports that can share the lane resources with the *west input port*. If the local input port is selected in *line 5* and the lane is labeled "orange" (see *line 6*), then the resulting configuration is shown in Figure 4d. It is obvious from this figure that no cycle exists in the lane as the path between the *west output port* and the *east input port* is not labeled. Thus, sharing the lane between the west input port and the local input port does not result in a deadlock configuration. The *lines 1 to 11* of Algorithm 1 are repeated until all the input ports are attached to a lane. Figure 5 shows a possible output design of the algorithm.

Switch links are used to switch between *primary* and *secondary* lanes, following the CDG corresponding to this deadlock-free *R-NoC*, provided in [22]. Data flows from input ports located on two distinct *primary* lanes cannot switch to the same *secondary* lane. Consider the generated topology in Figure 5, lane 2 is a *secondary* lane and is shared only with the west and local input ports since they both share *primary* lane 0. A similar argument holds for the south, east, and north input ports sharing lanes 1 and 3 buffering resources. Switch links can be inserted between the end of a *primary* lane and the beginning of a *secondary* lane. For instance in Figure 5, a switch link is added between the end of the *primary* lane L0 and the beginning of the *secondary* lane L2. In addition, switch links can be inserted at locations where multiple data flows contend for the lane resources, e.g., between the east and north input ports on lane L2. This enables data flows from the east to switch to the *secondary* lane when contention occurs between data flows from both ports. In Figure 5, *lane 0* hosts the local and west input ports, while *lane 1* hosts the east, south, and north input ports.

## 4. Implementation of *R-NoC* Router and NoC

We detail the synchronous elastic implementation of *R-NoC* routers. For illustration, we consider the design of a 4-lane *R-NoC* router similar to that described above.

### 4.1. Synchronous-Elastic Design Style

We so far assumed packet travel across lanes in a pipeline fashion and are thereby buffered. Whenever a packet cannot move forward anymore the entire pipeline must be stalled until the forward path is available. This control flow question is in conventional NoC routers tackled by FIFO queues which track their internal usage and perform handshaking with upstream/downstream FIFO queues. This obviously does not apply to R-NoC lanes which are similar to plain shift registers. Elastic Buffers [23] provide an elegant answer for that situation with a semi-asynchronous functioning in which handshaking is performed from pipeline stages. Several different flavors exist, either using Latches or Flip-Flops.

**Figure 6:**
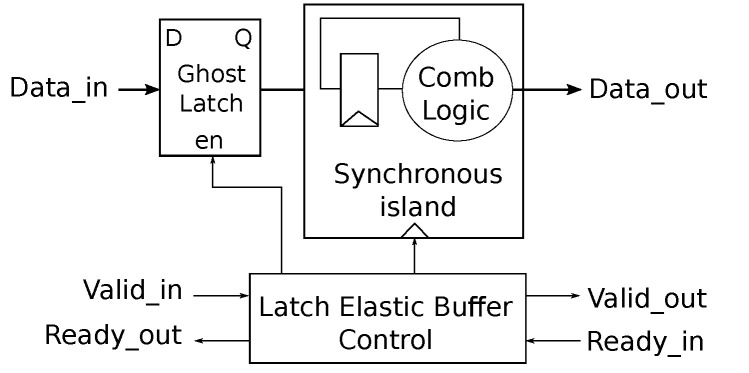
Principe of Elasticization of synchronous circuits.

Figure 6 illustrates the chosen elasticization approach chosen in which some logic is wrapped around a synchronous logic island. The elastic buffer control is a simple finite state machine that performs handshaking with up- and down-stream units, latches incoming data in a ghost latch, and issues clock edges to the synchronous island whenever needed for sampling data out of the ghost latch. In our particular case the synchronous island is a mere 32-bit register; note that the ghost latch incurs no additional latency as its role is confined to pipeline stalls/back-pressure.

### 4.2. R-NoC Topology

**Figure 7:**
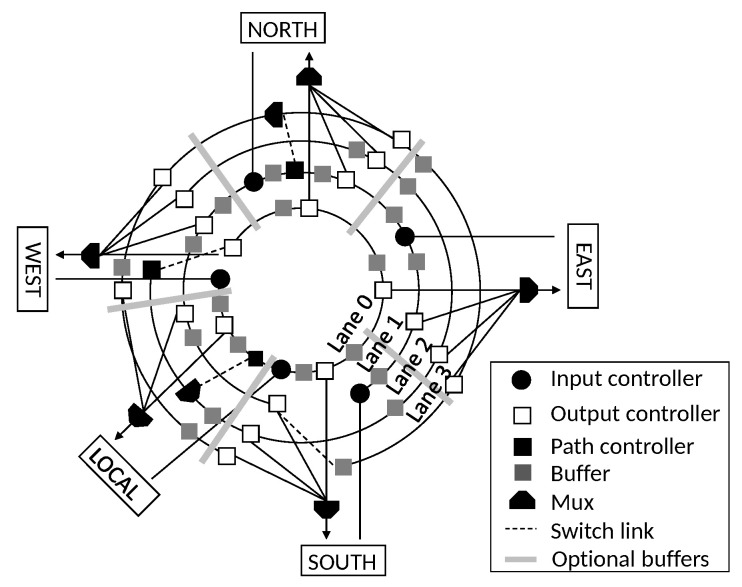
A 4-lane *R-NoC* topology.

Let us start from the implementation of a *R-NoC* topology with 4 lanes depicted in Figure 7. It consists of several sub-blocks such as controllers (input/output/path), buffers, and multiplexers. The buffers are distributed on the lanes as shown in Figure 7. Thus, packets from several input ports can exploit the buffering resources on the lanes for performance benefits. Basically, when a packet enters the router, it is received by the input controller. Route computation and packet output port encoding take place here before the packet is forwarded to the lane. The path controller switches the packet to a *secondary* lane if its path is blocked, while the output controller forwards a packet to the router output port if destined for the corresponding output, hence it is forwarded to the lane. *R-NoC* provides a highly distributed and adaptive topology allowing for additional lanes and ports to be added without incurring an increase in arbitration, and area/power overhead. Next, we detail the *R-NoC* router implementation.

### 4.3. Lane Pipeline

**Figure 8:**
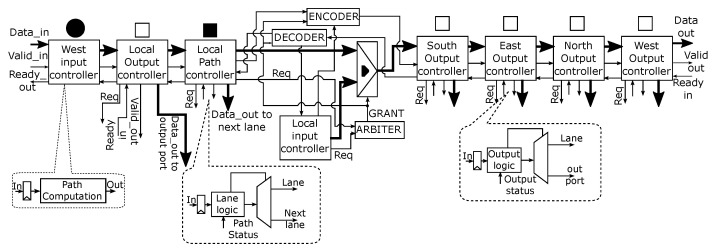
Implementation pipeline for lane 0.

The entire pipeline for *lane 0* is shown in Figure 8. The associated blocks, i.e., input, output, and lane controllers, make up the individual pipeline stages and communicate via *ready/valid* handshake protocol. Markers standing above each block share a similar meaning with those in Figure 7. Each block incurs only a clock cycle latency each, hence the pipeline is only *6 clock cycles* for the longest path on the lane, in other words, from west input port to north output port.

### 4.4. Input Controller

The input controller block consists of an Elastic Buffer (EB) and a *path computation* (PC) block. This is depicted in Figure 8. Detailed implementation of the EB block will be found in [22], area overhead of this block is minimal with a single clock-cycle latency. When a flit arrives at the input port of the router, it is forwarded to the PC block. The PC block is responsible for computing the packet output port. If the flit is a header-flit, the PC block decodes the packet destination address encoded in the flit and uses this information to compute the packet output port in the current router. The output port routing information is encoded in the header-flit of the packet before it is transmitted. The other flits follow the path already reserved by the header-flit since only the header-flit contains the routing information.

### 4.5. Output and Path Controller

The output controller is also displayed in Figure 8. It consists of an EB, an *output logic* block, and a *Demux*. When a flit is received, it is forwarded to the output logic block. Depending on the packet output information encoded in the packet header and the output status, the block selects one of two possible paths to forward the packet. The possibilities are as follows:

1. output port address matches and output port is free;
2. output port address matches but output port is busy and is not *local*;
3. output port address matches but output port is busy and is *local*;
4. output port address does not match;
5. output port address matches and output port controller is located on the *secondary* lanes *Lane 2* or *Lane 3* shown in Figure 7.

For cases (1), (3), and (5) the path labeled “out port” is selected and the flit is forwarded to the output port. However, the flit is forwarded to the lane for case (3) if the output port controller is located on *lane 1* (see Figure 7). For cases (2) and (4), the path labeled “Lane” is selected and the flit is forwarded to the lane. The reserved path is kept active for the other flits transmission. The *path controller* is similar to the output controller in terms of functionality. It forwards a packet to the lane if the packet path is not blocked on the *primary* lane. Otherwise, it switches the packet to a *secondary* lane. In Figure 7, the job of the path controller on lane 2-west is only to request the next router resource.

### 4.6. Arbitration

**Figure 9:**
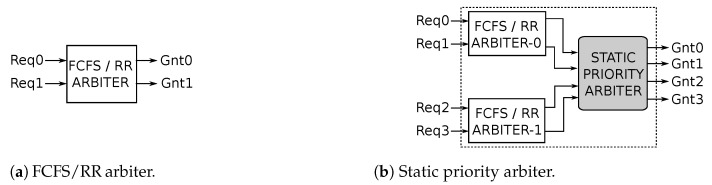
First-Come-First-Serve (FCFS)/Round-Robin (RR) and static-priority arbiter (**a**,**b**).

An arbiter is required to control access to shared network resources. Two kinds of arbiters are used in *R-NoC* to control access to lane buffers and output ports. A *2 input-request* arbiter is used to control access to shared lane resources as displayed in Figure 9a. This arbiter operates in two modes: *First-Come-First-Serve (FCFS)* mode for sequential requests and *Round-Robin (RR)* mode for simultaneous requests.

On the other hand, a static priority arbiter is used to control access to *R-NoC* output port as shown in Figure 9b. It is composed of two FCFS and RR arbiters, combined with a *static priority arbiter* that grants the output port request based on lane *priority*. The *FCFS/RR arbiter-0* arbitrates between a request coming from two *primary* lanes (lane 0 and lane 1), while the *FCFS/RR arbiter-1* arbitrates between a request coming from two *secondary* lanes (lane 2 and lane 3) using policies described previously. As shown in Figure 9b, the outputs of the two *FCFS/RR* arbiters are fed into the *static priority arbiter*. Note that only two inputs to the *static priority arbiter* can be asserted simultaneously: one output from *FCFS/RR arbiter-0* and the other from *FCFS/RR arbiter-1*. The output port is granted depending on the priority of the request. A request from *FCFS/RR arbiter-1* is given priority over a request from *FCFS/RR arbiter-0*, when simultaneously requests occur. This is because a request from *FCFS/RR arbiter-1* represents an output port request from a controller located on the *secondary* lane. The arbiters are implemented using Finite State Machines (FSM) with a combinational output (Mealy FSM), hence they can process requests as soon as they arrive without waiting for a clock edge.

The starvation of packets is an important concern in NoC design. It occurs when packets are constantly being denied network resources, i.e., connecting links and output ports. An example is a situation where low-priority packets are denied network resources because the resources are constantly being occupied by higher-priority packets. *R-NoC* arbitration/priority schemes are designed to avoid starvation of packets on lower-priority lanes. This is mainly due to *lane-switching* in the router which enables the switching of packets from lower to higher priority lanes. Intuitively, packet priorities are transient in *R-NoC* which helps to prevent starvation of packets.

### 4.7. R-NoC Output Port Block

**Figure 10:**
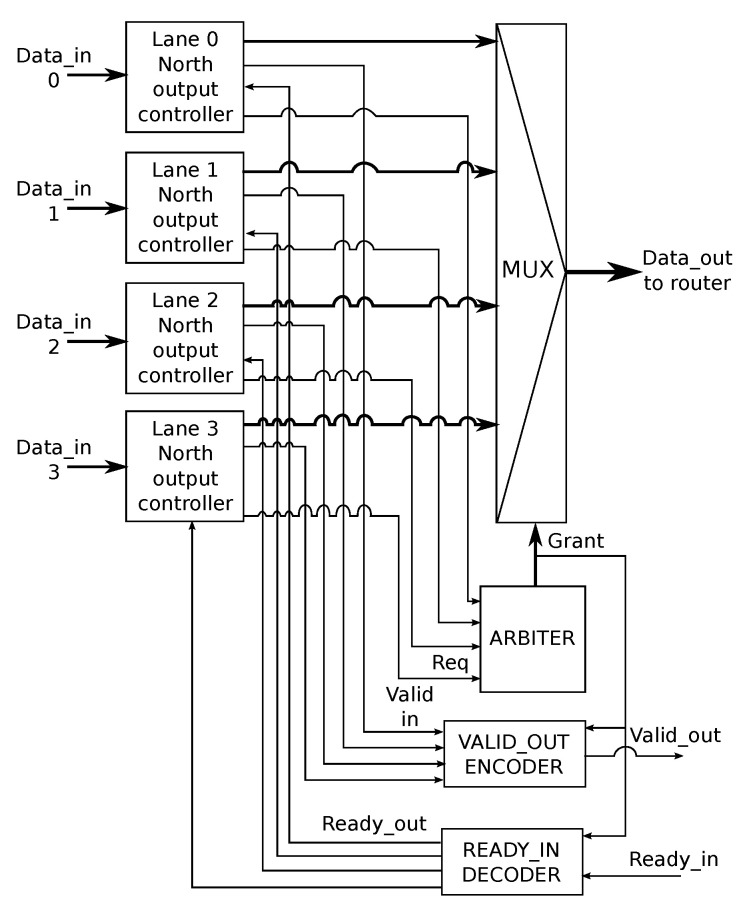
*R-NoC output port block*.

Figure 10 shows the *R-NoC north output port block*, which is typical of all the output ports in the router. The block receives data flows from each north output controller located on lane 0 to lane 3. As shown in Figure 10, the *grant* signal from the *Arbiter* is used to *select* one of the four data-flows that are inputs to the *Mux*. The selected data is forwarded to the *input controller* of the adjacent router. Remembering that each block communicates using the *ready/valid* handshake protocol, the output port block must ensure the following for the correct functioning of the circuit:

- only the *valid* signal of the output controller for which access has been granted is sent to the receiver.
- the receiver’s status, indicated by the *"ready_in"* signal, is communicated only to the output controller that has been granted access to the output port.

Thus, an *Encoder* is used to generate the correct *valid_out* signals, while the correct *ready_out* signals are generated using a *Decoder* as shown in Figure 10. A possible implementations of the blocks can be found in [24].

## 5. *R-NoC* Evaluation

Fairly benchmarking communication networks is a rather tedious and time-consuming process that requires long simulation times for properly assessing the interconnect behavior. Analytical models are a viable alternative for conventional NoC designs, and recent contributions [25] have even shown the opportunity of combining analytical models with simulations. Yet given the unconventional nature of *R-NoC*, we perform RTL and post-synthesis gate-level simulations for unbiased performance assessment. All results described in this section are gathered using *Cadence RTL Compiler* targeting a 45 nm CMOS cell library. Power results are obtained with *Synopsys PrimeTime PX* from the execution traces (VCD files) extracted from the simulated netlists. We evaluate the performance and power consumption of 6 different *R-NoC* router configurations. These 6 configurations are benchmarked and compared against the Hermes router whose RTL implementation is available and used in the sequel. *R-NoC* router area and power evaluations have been provided in [22]. We here focus on network-level evaluation for all configurations for unbiased comparative analysis.

### 5.1. Topology Exploration

**Figure 11:**
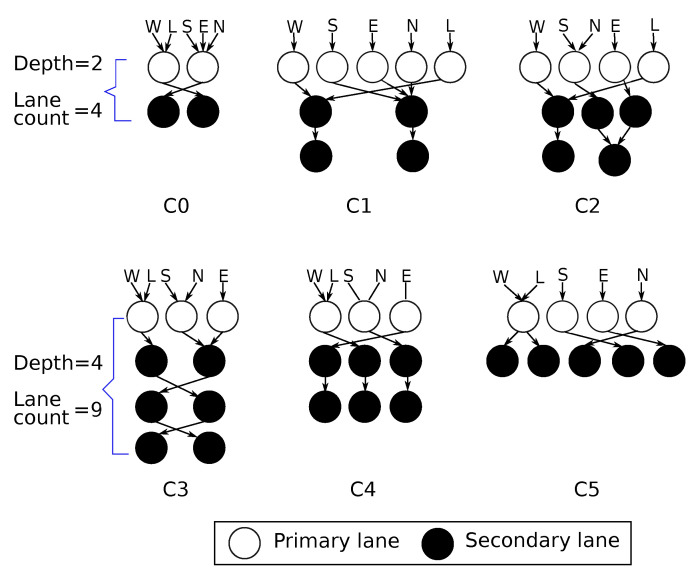
Considered *R-NoC* configurations.

Figure 11 gives the schematics of the considered *R-NoC* router configurations. Configuration *C0* is the 4 lanes version shown in Figure 7 with two *primary* lanes and two *secondary* lanes. This version cannot support five concurrent data flows supported by typical input-buffered routers such as Hermes [10]. The reason lies in the limited parallelism in its *primary* lanes (to which input ports are attached): packets may be blocked straight upon entering the router.

**Figure 12:**
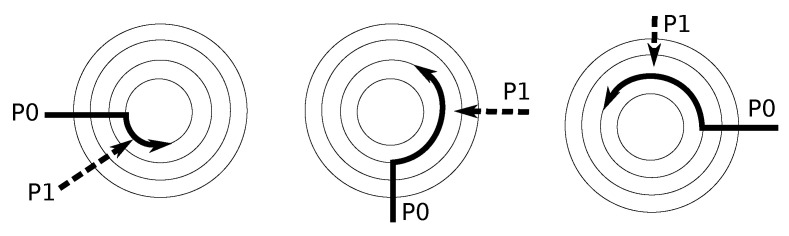
Blocked packet caused by different arrival times.

Three different scenarios illustrate this behavior in Figure 12, where a packet *P1* with a later arrival time is temporarily blocked because the lane is occupied by an earlier packet *P0*. In this scenario, the switch links are not available for use by the blocked packets. This situation occurs because the parallelism level on the *primary* lanes is limited. Therefore the router cannot always support five concurrent data flows supported in typical input buffered routers [22].

For ensuring fair comparisons across topologies we arbitrarily set the total number of lanes in each remaining configuration (i.e., C1 to C5) to 9. Table 2 shows the properties of the different 9-lane versions of *R-NoC*. Here, the configuration *C0* corresponds to the 4-lane architecture described in Figure 7, while the remaining configurations are 9-lane router designs.

*R-NoC* routers can have a maximum of five *primary* lanes indicated by the *parallelism level* column in Table 2. A level of 5 means the router has maximum parallelism on the *primary* lanes, while the *secondary* lanes are shared among packets from input ports. Such a router, e.g., *C1* configuration behaves like a typical input-buffered router at low traffic, while the *secondary* lanes are exploited under medium and high traffic. Another parameter denoted *depth* relates to the lane connectivity: in a router of depth *D*, a packet can at most be routed on *D* lanes before leaving the router. For the performance exploration, we consider a 4×4-mesh network. We use a packet length of 10 flits, with flit size of 32 data bits in presented simulations. In our VHDL test benches, the processing blocks attached to the routers’ local ports serve as both producers and consumers of packets. For each tested injection rate, the simulation runs until a stable average latency is reached, at which point the value is recorded, and the next simulation is executed.

**Figure 13:**
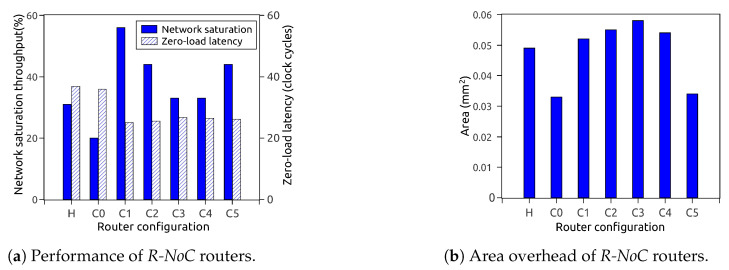
Performance and area-overhead of *R-NoC* routers (H denotes Hermes).

Figure 13a depicts the performance of different router configurations for uniform traffic patterns. It shows that having a high level of parallelism on *primary* lanes is rewarding, since it mitigates performance loss caused by temporarily blocked packets and avoids unnecessary contentions on the lanes. As displayed in Figure 13a, the zero-load packet latency is also improved for configurations with higher parallelism levels. Router configurations with a higher level of parallelism outperform those with less parallelism for similar or even lesser lane depth. Figure 13b shows the corresponding area overhead for the router configurations. It is observed that the performance of the routers does not solely depend on the area, but on their topological parameters.

#### 5.1.1. Performance for Different Traffic Patterns

**Figure 14:**
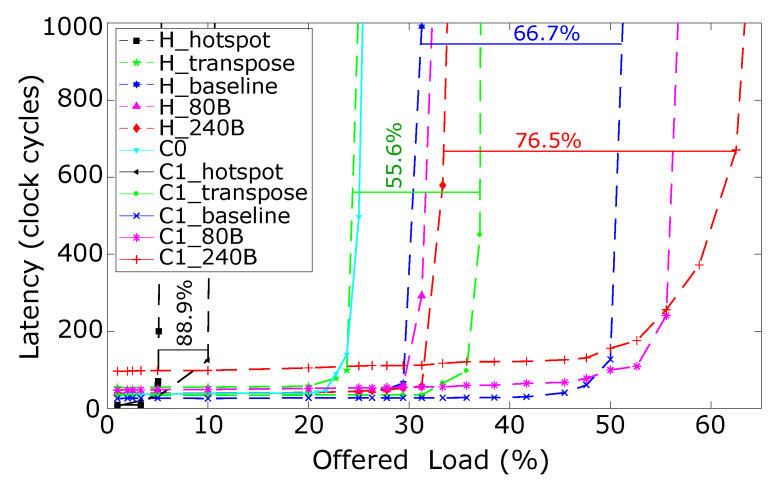
Performance comparison for *R-NoC* and Hermes routers. XB denotes baseline router with additional *X* buffers.

The *R-NoC* configuration *C1* is selected for comparing with Hermes [10]. Both routers have a buffer count of 80 buffer positions of 32 bits for a fair comparison. Similarly to *R-NoC*, the Hermes router uses *wormhole* flow-control and *XY-routing*. It employs *credit-based* buffer management scheme. Figure 14 shows the latency vs. offered load for both routers. We also display the performance of the *4 lanes R-NoC*. As shown in the plot, the Hermes router outperforms the *R-NoC C0* router, offering better network saturation throughput. As explained earlier, the Hermes router can always support up to five concurrent data-flows, for different source-destination pairs, regardless of the packet arrival time. Conversely, packets for different *source-destination* pairs may compete for channel resources in the *R-NoC C0* router. This motivated the *9-lane versions of R-NoC*. The 9-lane *C1_baseline* version provides a performance improvement of over 60% compared to the Hermes baseline router, denoted by *H_baseline*. In Figure 14, *R-NoC* with additional buffers significantly outperforms the Hermes routers (with additional buffers) by up 87%.

We simulated the baseline routers using *transpose and hotspot* traffic patterns. In transpose traffic pattern, each node communicates only with the destination node with the upper and lower halves of its own address. In hotspot traffic pattern, all nodes communicate with a specific node, referred to as *the hotspot* node. This creates a higher network contention when compared to the transpose and uniform traffic. It is observed that the network saturation throughput for *R-NoC* is improved by 61% and 88% for transpose and hotspot traffic respectively when compared to the Hermes router, which confirms the intrinsic ability of *R-NoC* to support specific traffic patterns by means of dynamically allocating buffer resources whenever needed.

#### 5.1.2. R-NoC Network-Level Power Consumption

**Figure 15:**
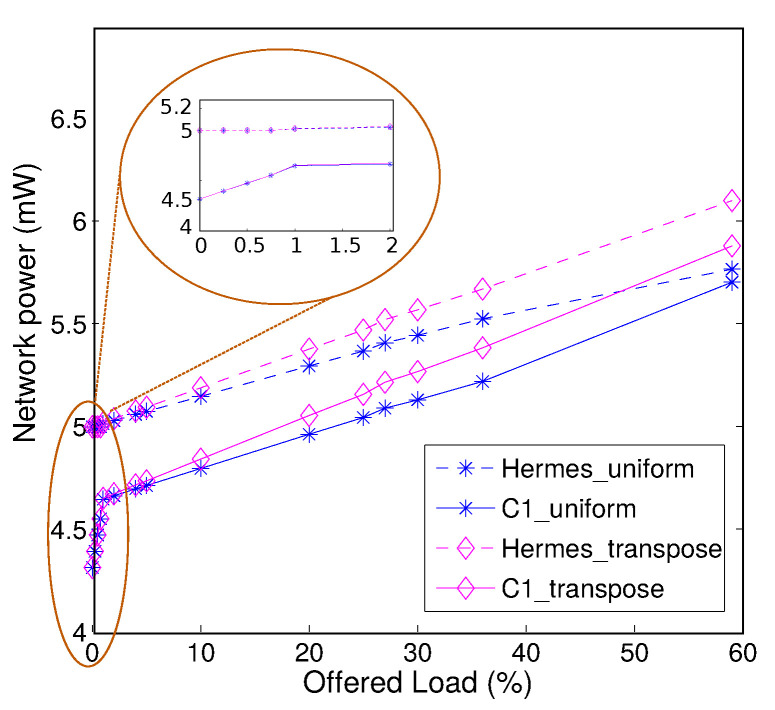
Network-level power consumption comparison.

The network-level power comparison of *R-NoC (C1)* and Hermes is shown in Figure 15. Power estimation is carried out at the gate level with a clock frequency of 300 MHz on the chosen 45 nm CMOS process. A network size of 2×2 (for simplicity reasons) is considered for uniform and transpose traffic patterns. It is visible from the plot that the *R-NoC* router achieves lesser power consumption than Hermes [10] for both traffic patterns. This is because packet travel across a *R-NoC* router overall incurs lesser switching activity, notably due to the absence of centralized arbiter and FIFO queues.

We observe that *R-NoC* consumes 15% lesser power compared to Hermes in idle mode (0% injection rate) and at very low injection rates This is due to its dynamic use of buffering resources, i.e., only primary lanes get used under these conditions. This makes *R-NoC* suitable for networks that operate at low injection rates [15] such as networks connecting *L1 caches* and *L2 cache banks* [26].

#### 5.1.3. Scalability for Different Network Sizes

**Figure 16:**
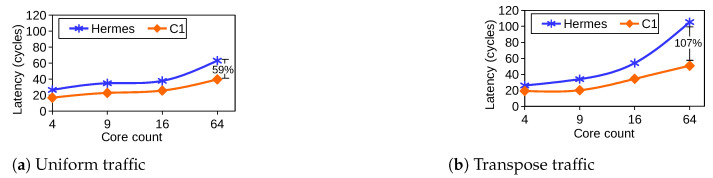
Scalability comparison of *R-NoC C1* with Hermes [10]. 4: 2 × 2 mesh; 9: 3 × 3 mesh, 16: 4 × 4 mesh, 64: 8 × 8 mesh network. Latency: average network zero-load latency.

We assess the scalability *R-NoC* according to different network sizes under the zero-load latency perspective. A comparison with Hermes is shown in Figure 16, while selecting *R-NoC (C1)* and Hermes [10] with uniform and transpose NoC traffics. We observe that *R-NoC (C1)* scales better than Hermes. The latter incurs high latency (almost 2× increase) when the network size grows from 16 cores to 64 cores for both traffic patterns. This makes the Hermes router less suitable compared to *R-NoC (C1)* for latency-sensitive NoC applications such as cache coherent NoCs where minimal latency is crucial for cache miss traffics.

Note that in our preliminary study [22], we also showed that *R-NoC* offers better performance improvement than Hermes when increasing the number of buffers.

#### 5.1.4. Application Performance

We estimate *R-NoC* performance for realistic applications. Table 3 gives the properties of these applications in terms of task count. The applications are represented as task graphs, where inter-task communications require specific communication bandwidth for meeting application requirements.

**Figure 17:**
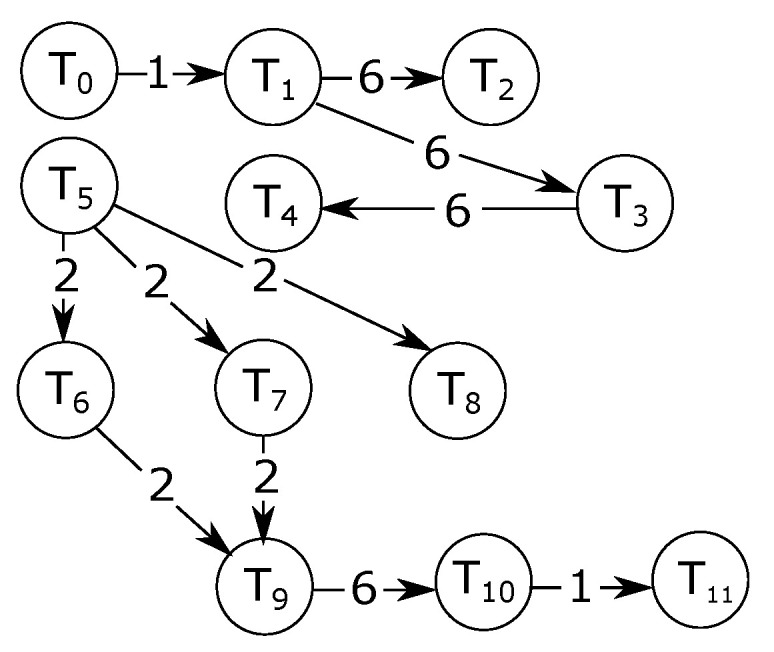
A sample app (inter-task required bandwidth in Mbps).

Figure 17 shows a sample application task graph. In the graph shown in Figure 17, the nodes represent tasks, while the edges represent communications. As an example, a bandwidth of 6 Mbps is required for the communication between task $T_{1}$ and task $T_{2}$. The method for generating the experimental traffic for these applications relies on [2].

**Figure 18:**
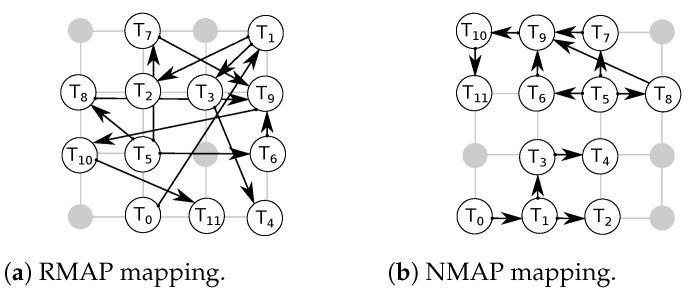
E3S random and near mapping scenarios.

We considered random (RMAP) and near (NMAP) mappings. In NMAP, communicating tasks are placed in close proximity to each other to reduce average packet latencies. We mapped the applications on 4×4 networks. Multiple tasks are mapped on a single core for applications with more task count than network node count. Examples of such applications include MMS and E3S telecom applications. For such applications, the task execution is performed sequentially. For example, if tasks $T_{1}$ and $T_{2}$ are mapped on the same core. Then, the execution begins with $T_{1}$ followed by $T_{2}$ and then back to $T_{1}$. Figure 18a and Figure 18b respectively show the RMAP and NMAP mappings of the sample application depicted in Figure 17.

**Figure 19:**
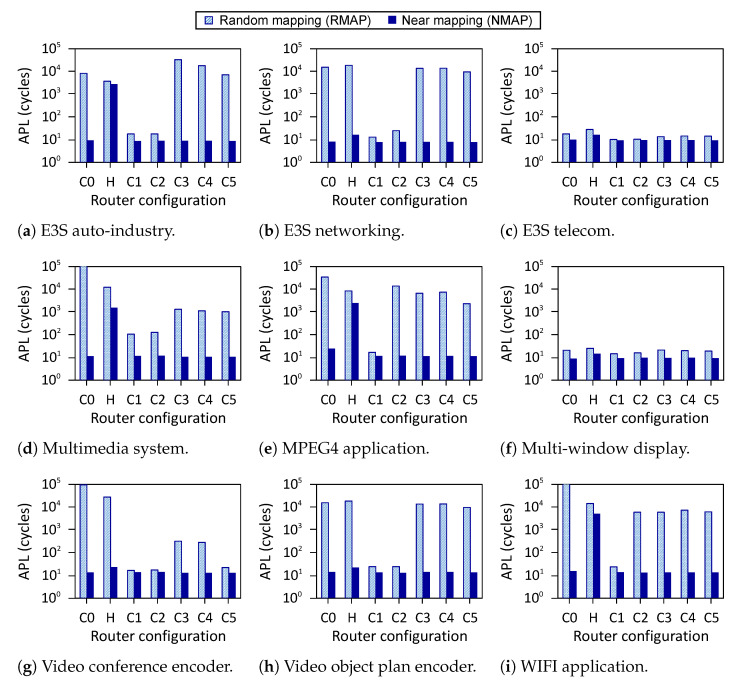
Performance for application. APL: Average packet latency. *Y-axis* uses log scale (**a**–**i**).

Figure 19 shows performance results of the applications for various *R-NoC* configurations in which we report the average communication latency for both RMAP and NMAP. We observe that Hermes provides improved performance for most of the applications compared to *R-NoC C0* for RMAP mapping, due to the reasons explained in Section 5.1.1. This is again due to the limited parallelism level in *R-NoC C0* compared to Hermes. Hence, communication bottleneck increases in *R-NoC C0* for RMAP, which in turn leads to an increase in packet latency. However, *R-NoC C0* provides better performance compared to Hermes for NMAP mappings, and this for all of the applications. This is due to the shorter router pipeline in *R-NoC C0* compared to Hermes. Thus, packets in *R-NoC C0* can be quickly routed to their desired destination. In addition, in case of contention in NMAP mapping (when multiple tasks communicate with one task, creating hotspots in a network), *R-NoC* can mitigate performance loss by dynamically allocating its buffering resources. This is not possible in Hermes where the buffers are pinned to the input ports.

The *R-NoC C1* design provides better performance compared to the other *R-NoC* configurations, especially for RMAP. This observation is similar to synthetic traffic patterns discussed earlier in this section. This is favored by the high-parallelism level of *R-NoC C1*. We also observe that the performance of the other *R-NoC* configurations for NMAP mapping is similar to that of *R-NoC C1*. This is because the network load remains modest for NMAP mapping. The *E3s telecom* and *Multi-window display* applications lead to overall lower network latency especially for RMAP compared to the other applications. An analysis of the application task graphs reveals that most of the inter-task communication for *E3s telecom* and *Multi-window display* applications occur at lower injection rates, which accounts for overall lower latency. Overall, the combination of resource sharing and adaptive features of *R-NoC* gives it an edge over Hermes [10].

## 6. Evaluation of *R-NoC* for Diagonally Linked Mesh NoCs

**Figure 20:**
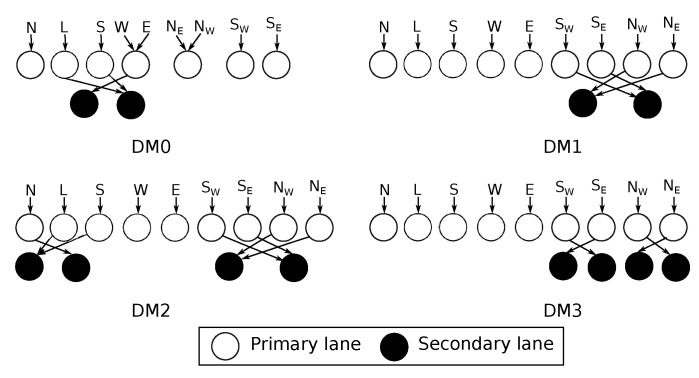
Considered *R-NoC-D* configurations.

The *R-NoC* router concept can be readily extended to support other network topologies, leveraging on its distributed control nature. We here design a diagonally linked mesh network topology, called *R-NoC-D*. This extension uses *quasi-minimal* adaptive routing proposed in [7], where priority is given to the diagonal links. The non-diagonal links are used if the desired diagonal link is not available. We exploit *R-NoC* highly-adaptable architecture to realize significantly higher performance for diagonally-linked mesh networks without corresponding area/power cost compared to typical input buffered routers. We explore different *R-NoC-D* configurations and investigate their performance/cost trade-offs. Figure 20 gives the schematics of these configurations, while Table 4 summarizes their characteristics together with the basic mesh router configuration C1, i.e., one of the best performing seen so far (schematic in Figure 11).

Router configurations with an equal number of ports and parallelism level have maximum parallelism on the *primary* lanes, while the *secondary* lanes are shared among packets from input ports. Such routers behave like a typical input buffered router at low traffic, while the *secondary* lanes are exploited at medium/high traffic. The main difference between *DM2* and *DM3* is in their *primary* lanes. In *DM3*, a *primary* lane (with diagonal input port) is reserved only for a diagonal output port, while non-diagonal output ports are also attached to *primary* lanes (with diagonal input ports) in *DM2*. We expect *DM3* to provide better performance since packets utilizing the diagonal output ports travel over shorter distances before reaching their destination.

**Figure 21:**
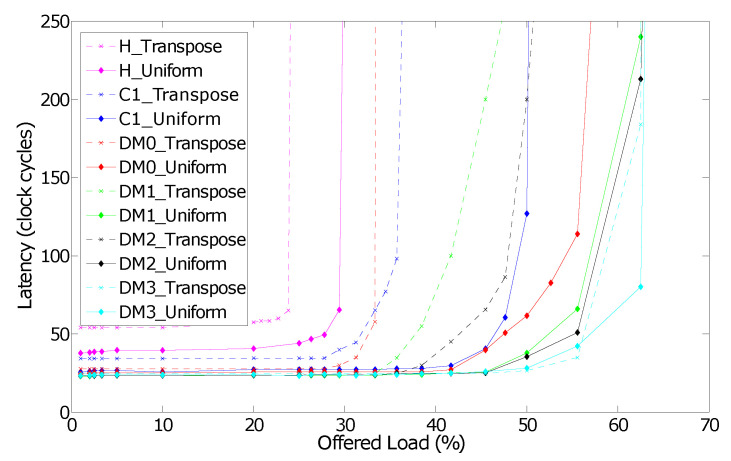
Performance of *R-NoC-D* routers (H for Hermes).

Figure 21 depicts the performance of different router configurations for uniform and transpose traffic patterns. The diagonal links indeed improve network performance for these traffic patterns since they provide shorter paths between network nodes and more communication links. The zero-load packet latency is also reduced with diagonal links. Assigning only the diagonal input and corresponding output ports to a lane further improves the network performance. For instance, it is the case of the *DM3* router configuration.

**Figure 22:**
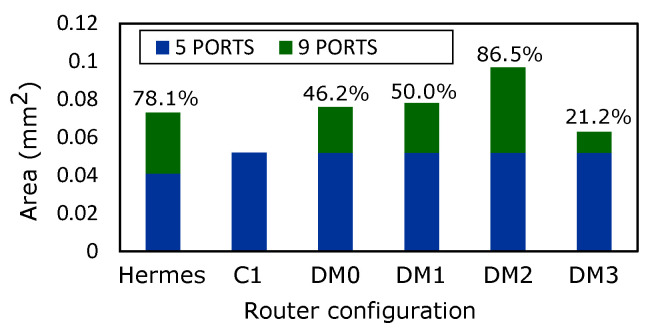
Router area: annotated percentages quantify the overheads introduced by additional ports.

Figure 22 presents the estimated area overhead corresponding to the routers. We display the cost incurred when additional ports are added to the router configuration. It is observed that adding additional ports in Hermes incurs massive costs compared to *DM0, DM1* and *DM3* routers. On the other hand, it is worth mentioning that the performance of the routers does not solely depend on the area, but on their topological parameters.

### 6.1. Performance Scaling through Buffer Insertion

To assess the scalability of *R-NoC-D*, we keep the same versions used previously as baselines. Additional buffers are then inserted and resulting designs are compared with their respective baseline versions. Note that buffers are evenly distributed on the lanes.

**Figure 23:**
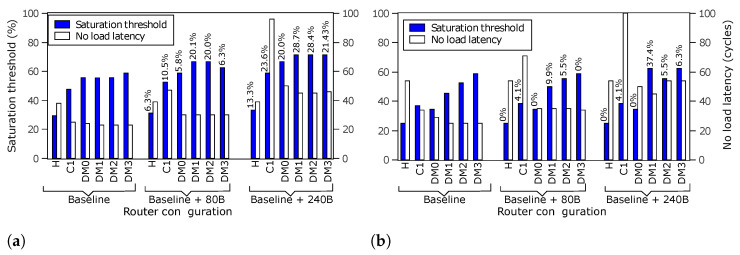
Performance for *R-NoC-D* and Hermes. Numbers on bars denote % increase in saturation threshold compared to baseline. (**a**) Performance for uniform traffic pattern. (**b**) Performance for transpose traffic pattern.

Figure 23 shows the performance of the routers with additional buffers, for uniform and transpose traffic patterns. The network saturation throughput is now increased. The router configurations with diagonal links provide better scalability. The figure also shows the corresponding performance of the Hermes router baseline and with additional buffers. As stated earlier, the Hermes baseline version has a similar configuration with *R-NoC (C1)* for a fair comparison. We observe that *R-NoC-D* configurations provide improved network saturation thresholds compared to Hermes for both uniform and non-uniform traffic patterns. Configuration *C1* incurs a significant increase in zero-load latency when the buffer count on a lane increases significantly, e.g., additional 240 buffers in Figure 23. This is due to the lack of *bypass logic* in the elastic buffer pipeline assembled on the lane. Thus, each elastic buffer incurs a clock cycle even at low traffic injection rates.

The *DM3* configuration is selected for comparison with Hermes [10]. Table 5 indicates the area evaluation for both routers. The area overhead *R-NoC-D (DM3)* is 13.7% less than that of Hermes for a similar number of ports. This supports our claim that *R-NoC* can be extended to provide significant performance improvement at an effective cost due to its highly adaptable architecture. We also display in Table 5 the power results for both the shortest (i.e., Min power) and the longest (i.e., Max power) path on a given lane in *DM3*. The power results were obtained for a single link operation when the routers were operated at a similar frequency. *R-NoC-D* provides power reduced of 9% over Hermes for the short path.

### 6.2. R-NoC-D Network-Level Power Consumption

Similar to *R-NoC*, we carry out power estimation of *R-NoC-D* at Gate level using *Synopsys PrimeTime PX* and the 45 nm CMOS cell library, at 300 MHz clock frequency. For the sake of simplicity, a network size of 2×2 and the transpose traffic pattern were considered in reported simulations.

**Figure 24:**
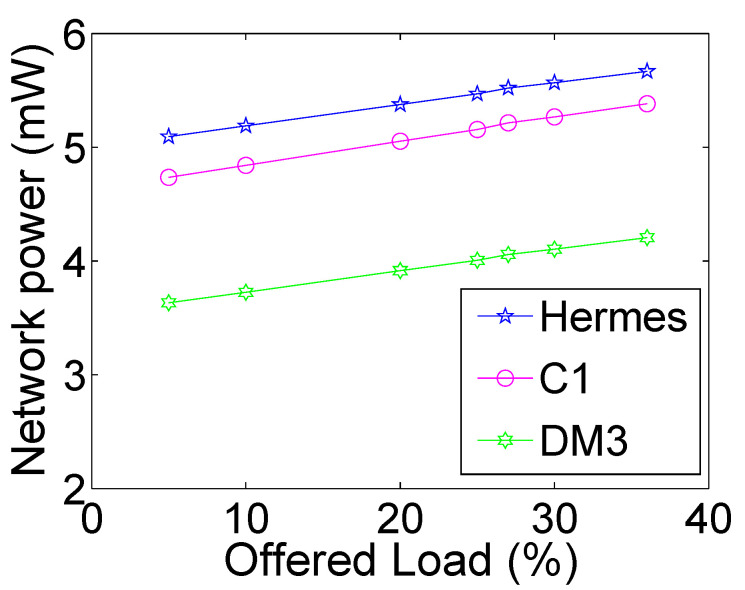
Network-level power consumption comparison.

Figure 24 depicts the power comparison of the selected routers. The performance improvements enabled by *R-NoC and R-NoC-D* routers over Hermes are not achieved at the expense of higher power consumption. Here, both *R-NoC and R-NoC-D* achieve lesser power consumption than Hermes thanks to their faster data transmission, which leads to reduced network switching activities in the routers. *R-NoC-D* provides the lowest power consumption because of the shorter network diameter and therefore lesser average number of hops between routers.

### 6.3. Comparison with Existing Solutions

Beyond Hermes, we compare *R-NoC* and *R-NoC-D* with Rotary [6] and single cycle routers, which are also relevant given the design features they share with *R-NoC*.

#### 6.3.1. Comparison with Rotary

Table 6 shows the comparison of Rotary [6] and *R-NoC-D (DM3)*. The head-flit travel time for *R-NoC-D* is twice less than that of Rotary [6] since it uses a shorter router pipeline requiring only two cycles. Table 6 also displays the network saturation for both routers for similar network sizes. The network saturation threshold for Rotary outperforms that of R-NoC. However, this performance comes at expense of large area/power overhead associated with large buffers; the Rotary version for which performance figures are reported in Table 6 indeed features well over 10kB of buffer memory. The Rotary router indeed [6] uses combined VCT and bubble flow-control, i.e., local bubble scheme, which leads to poor buffer utilization especially for cache-coherent traffics. The reason is that the majority of such traffics are short packets, typically single-flit packets. The implication is that short packets must also be regarded as long packets which leads to poor buffer utilization [34].

#### 6.3.2. Comparison with VC and Single Cycle Routers

Finally, we compare the performance of *R-NoC* router w.r.t. state-of-the-art virtual channel (VC) based routers, while considering the same mesh network topology and the same buffer count.

Table 7 reports the main results. The *BIVR* [16] router represents a typical VC router with 5 pipeline stages, while the *IVR-SC* [35] and *FOVR-LS* [36] are *single cycle routers* requiring only *one* clock cycle for a single flit to travel-through the router. In general, the network zero-load latency for *FOVR-LS* is lower than that of *C1 and DM3* for equal packet length, while the network zero-load average latency for *IVR-SC* is only marginally lower than that of *C1 and DM3*. The interesting observation is that the network saturation threshold for *C1* is highly competitive compared to that of the VC routers, while *DM3* provides better network performance due to its internal topology and shorter network links.

## 7. Conclusions and Perspectives

We have presented an extensive evaluation of *R-NoC*, a highly customizable NoC router template with inherent and effective resource sharing. *R-NoC* consists of multiple lanes shared by input/output ports to maximize resource utilization for performance and energy benefits. *R-NoC* is implemented based on 45 nm CMOS and benchmarked against Hermes [10], which is a typical input-buffered router. *R-NoC* is shown to significantly improve network performance, provide enhanced scalability, and consume less power compared to typical input-buffered routers. Thus, the dynamic resource allocation and resource sharing features of *R-NoC* give it an edge over typical input-buffered routers. To improve network performance, *R-NoC* has been extended to provide for diagonally-linked mesh network topology, referred to as *R-NoC-D*. This extension improves performance compared to its mesh counterparts. Contrary to input buffered routers, the performance improvement of *R-NoC-D* comes at a reduced area/power cost. All of the presented *R-NoC* topologies can be devised thanks to a construction algorithm also presented in this paper, that makes it possible to generate valid topologies based on input parameters.

Our short-term perspectives include the evaluation of performance and energy gains on shared-memory, latency-sensitive, computer machines with benchmark suites such as PARSEC. The current versions of *R-NoC* and *R-NoC-D* work in best-effort mode. Future work aims at extending the routers to provide QoS in terms of guaranteed packet throughput and latency through the use of virtual channels (VCs). We also plan to explore further *R-NoC* architectures for performance improvements, while devising smart power management techniques capable of aggressive power reduction at low traffic injection rates. Finally, *R-NoC* is a highly parameterizable NoC template that opens interesting design-space exploration questions. Beyond the obvious investigations on the properties of the router-internal topologies, some other parameters such as uneven buffer distribution, or heterogeneous NoC design via position-specific custom router topologies are also some interesting research directions we wish to investigate.

## Figures and Tables

**Table 1:** *XY Routing* valid source and destinations.

Source (Input Ports)	Destinations (Outputs Ports)
Local	West, South, East, North
West	Local, South, East, North
East	West, South, Local, North
North	South, Local
South	North, Local

**Table 2:** *R-NoC* configurations (P/S denotes the ratio of *primary* over *secondary* lanes).

Config.	Lane Depth	Parallelism Level	P/S (%)	No. of Lanes
C0	2	2	50	4
C1	3	5	56	9
C2	3	4	44	
C3	4	3	33	
C4	3	3	33	
C5	2	3	44	

**Table 3:** Realistic application characteristics.

Applications	Number of Tasks
E3S auto-indust (E3S-AUTO) [27]	24
E3S networking (E3S-NET) [27]	12
E3S telecom (E3S-TEL) [27]	30
Multimedia system (MMS) [28]	25
MPEG4 application (MPEG4) [29]	12
Multi-window display (MWD) [30]	12
Video conference encoder (VCE) [31]	25
Video object plan encoder (VOPD) [32]	16
Wifi application (WIFI) [33]	20

**Table 4:** Router configurations.

Config.	No. of Ports	Parallelism Level	No. of Lanes
C1	5	5	9
DM0	9	5	9
DM1		9	11
DM2		9	13
DM3		**9**	**13**

**Table 5:** Comparison of Hermes and *R-NoC-D* routers.

Router	Area (mm${}^{2}$)	Power (mW)	
Hermes (9 ports)	0.073	3.6	
*R-NoC-D (DM3)*	0.063	Min	Max
		3.3	4.0

**Table 6:** Comparison with Rotary.

Router	Head-Flit	Saturation	Flow-Control	Network
	(Cycles)	(%)		Topology
Rotary [6]	4	75	VCT/bubble	2D-Torus
**C1**	**2**	**50**	**Wormhole**	**Mesh**
**DM3**		**63**		**DMesh**

**Table 7:** Network saturation throughput (%) of virtual-channel (VC) based routers and *R-NoC-D* for uniform traffic pattern. Pck_L: Packet length.

Pck_L	RIVR [16]	IVR-SC [35]	FOVR-LS [36]	C1	*DM3*
4	70%	71%	61%	57%	**80%**
8	54%	56%	53%	53%	**67%**
12	48%	49%	51%	50%	**57%**
16	45%	46%	47%	47%	**55%**

## Data Availability

This research work relies on datasets and sources that are publicly available and listed below:
HERMES - https://www.inf.pucrs.br/~calazans/research/Projects/Hermes/Hermes.html E3S - http://ziyang.eecs.umich.edu/~dickrp/e3s/
